# Mentalization for Offending Adult Males (MOAM): study protocol for a randomized controlled trial to evaluate mentalization-based treatment for antisocial personality disorder in male offenders on community probation

**DOI:** 10.1186/s13063-020-04896-w

**Published:** 2020-12-07

**Authors:** Peter Fonagy, Jessica Yakeley, Tessa Gardner, Elizabeth Simes, Mary McMurran, Paul Moran, Mike Crawford, Alison Frater, Barbara Barrett, Angus Cameron, James Wason, Stephen Pilling, Stephen Butler, Anthony Bateman

**Affiliations:** 1 Research Department of Clinical, Educational and Health Psychology, University College London, London, UK; 2 Anna Freud National Centre for Children and Families, London, UK; 3 Portman Clinic, Tavistock and Portman NHS Foundation Trust, London, UK; 4 Institute of Mental Health, University of Nottingham, Nottingham, UK; 5 Centre for Academic Mental Health, Population Health Sciences Department, Bristol Medical School, University of Bristol, Bristol, UK; 6 Centre for Mental Health, Imperial College, London, UK; 7 School of Law, Royal Holloway, University of London, London, UK; 8 Institute of Psychiatry, Psychology and Neuroscience, King’s College London, London, UK; 9 National Probation Service London Division, London, UK; 10 Population Health Sciences Institute, Newcastle University, Newcastle upon Tyne, UK; 11 MRC Biostatistics Unit, University of Cambridge, Cambridge, UK; 12 Psychology Department, University of Prince Edward Island, Charlottetown, Canada

**Keywords:** Antisocial personality disorder, Randomized controlled trial, Mentalization-based treatment, Probation, Personality disorder, Offenders, Aggression, Violence, Patient and public involvement, Economic evaluation

## Abstract

**Background:**

Antisocial personality disorder (ASPD), although associated with very significant health and social burden, is an under-researched mental disorder for which clinically effective and cost-effective treatment methods are urgently needed. No intervention has been established for prevention or as the treatment of choice for this disorder. Mentalization-based treatment (MBT) is a psychotherapeutic treatment that has shown some promising preliminary results for reducing personality disorder symptomatology by specifically targeting the ability to recognize and understand the mental states of oneself and others, an ability that is compromised in people with ASPD. This paper describes the protocol of a multi-site RCT designed to test the effectiveness and cost-effectiveness of MBT for reducing aggression and alleviating the wider symptoms of ASPD in male offenders subject to probation supervision who fulfil diagnostic criteria for ASPD.

**Methods:**

Three hundred and two participants recruited from a pool of offenders subject to statutory supervision by the National Probation Service at 13 sites across the UK will be randomized on a 1:1 basis to 12 months of probation plus MBT or standard probation as usual, with follow-up to 24 months post-randomization. The primary outcome is frequency of aggressive antisocial behaviour as assessed by the Overt Aggression Scale – Modified. Secondary outcomes include violence, offending rates, alcohol use, drug use, mental health status, quality of life, and total service use costs. Data will be gathered from police and criminal justice databases, NHS record linkage, and interviews and self-report measures administered to participants. Primary analysis will be on an intent-to-treat basis; per-protocol analysis will be undertaken as secondary analysis. The primary outcome will be analysed using hierarchical mixed-effects linear regression. Secondary outcomes will be analysed using mixed-effects linear regression, mixed-effects logistic regression, and mixed-effects Poisson models for secondary outcomes depending on whether the outcome is continuous, binary, or count data. A cost-effectiveness and cost-utility analysis will be undertaken.

**Discussion:**

This definitive, national, multi-site trial is of sufficient size to evaluate MBT to inform policymakers, service commissioners, clinicians, and service users about its potential to treat offenders with ASPD and the likely impact on the population at risk.

**Trial registration:**

ISRCTN 32309003. Registered on 8 April 2016.

## Background

Personality disorder is substantially overrepresented in offending populations. Multiple studies consistently report a high prevalence of personality disorder in offenders in general [1] and in individuals with convictions for violent offences in particular [2]. Antisocial personality disorder (ASPD) is the most common personality disorder in criminal justice settings [3, 4]. This disorder is characterized by a failure to conform to social norms with respect to lawful behaviours; irritability and aggressiveness; impulsiveness; disregard for the feelings and safety of others; and disregard for one’s safety and for the consequences of one’s behaviour [5, 6]. The aim of this study is to establish if an intervention targeting the spectrum of symptoms and behaviours associated with ASPD is of value in improving individual mental health and reducing associated public health and social burden.

ASPD is associated with high levels of comorbid conditions [7–10]. Over 90% of individuals with ASPD have at least one other psychiatric disorder [11], at least 50% have co-occurring anxiety disorders [12], and 25% have a depressive disorder [13]. Men with ASPD are 3–5 times as likely to misuse alcohol and illicit drugs as those without ASPD [14], and women with ASPD have an even higher likelihood of drug and alcohol misuse [14, 15]. ASPD is also associated with physical disability [16] and premature mortality [7]. Men with ASPD have a higher rate of premature death than men of the same age without the disorder, due not only to an increased risk of suicide but also to reckless behaviours such as drug misuse and aggression [8].

The prevalence of ASPD has been reported as 0.6% in the UK general population [5], although it may be underdiagnosed in the community [17]. Nonetheless, there is a wide disparity between the prevalence of ASPD among the general population and among the offending population: in the UK prison population, just under two thirds of male remand prisoners, half of male sentenced prisoners, and one third of female prisoners meet diagnostic criteria for ASPD [4]. Although the prevalence of ASPD in probation services has not been fully explored, a screening study showed that nearly half of those on probation had probable personality disorder [18], an estimate likely to be higher among those supervised as ‘high risk’. In addition, reforms to the UK probation system in 2013 mean that ASPD is likely to be even more prevalent in the high-risk population supervised under the National Probation Service (NPS) in which the study is being conducted.

The contribution of ASPD to violent criminal behaviour is clear: it is associated with a significantly increased likelihood of committing violent crimes [19, 20] and is highly predictive of future violence, future reconviction or rapid re-incarceration upon release, and severity of recidivism [21, 22]. The social impact of individuals whose ASPD manifests in the form of violent criminal behaviour includes direct physical and emotional harm to victims, damage to property, use of police time, involvement with the criminal justice system, and increased use of healthcare facilities [23]. Finding an effective treatment to reduce aggression in individuals with ASPD has potential public health benefits, both directly, by improving the health of those with ASPD, and indirectly, through reducing the adverse consequences for victims, families, and communities.

Although the treatment of ASPD is a well-recognized priority, many studies evaluating interventions for antisocial behaviour have not looked at personality disorder diagnoses, and no intervention has been established as the treatment of choice for addressing the symptoms of ASPD. The paucity of studies in this area is notable [24]; only a small number of high-quality trials of specific therapies for ASPD have been conducted. We conducted a systematic literature search under National Collaborating Centre for Mental Health guidance, focusing on papers published from 2009 to 2016 (since the UK National Institute for Health and Clinical Excellence (NICE) review [5] was completed), using a strategy consisting of randomized controlled trial (RCT) and systematic review filters combined with subject heading and free-text phrases for ASPD. The search strategy was run using the standard mental health-related and allied health bibliographic databases (PsycINFO, EMBASE, AMED, and MEDLINE—using the Ovid interface). The search yielded 460 results after deduplication. After excluding results that were not journal papers (11), did not focus on ASPD (301), did not focus on quantitative treatment outcomes for existing ASPD (133), and did not differentiate ASPD from other personality disorders (10), only five results remained: three trials [25–27] and two Cochrane reviews [28, 29]. Of the trials, only one study was conducted in a community sample [25]; this was not a definitive RCT but an exploratory trial in a small sample of adult men with ASPD (*N* = 52), which investigated the feasibility of carrying out a full RCT of cognitive-behavioural therapy versus treatment as usual. Four ongoing studies were also found by searching trial registries. These trials’ sample sizes were relatively small: one of *N* = 114, the others of *N* < 50. Moreover, within their samples, these trials did not differentiate ASPD from other disorders such as borderline personality disorder (BPD) or paranoid personality disorder, or from autism. The search also revealed a number of meta-analyses, including a recent Cochrane review, which highlight the lack of evidence to support the use of any intervention for ASPD and urgently recommend that research be carried out to determine effective and cost-effective interventions [5, 28–31]. In this paper, we present the protocol for a large-scale RCT to generate new knowledge to address this gap in existing knowledge.

We propose the randomized evaluation of a psychotherapeutic model, mentalization-based treatment (MBT) for ASPD (MBT-ASPD). MBT is a manualized intervention that is based on the assumption that individual differences in the capacity to understand behaviour in terms of mental states arise as a result of variability in the social environment in early childhood, when mental state understanding of behaviour is normally acquired [32–34]. MBT integrates cognitive and relational components and provides a structured therapeutic process during which the patient’s mind becomes the focus of treatment, centring on his/her capacity to accurately interpret the meaning of actions in terms of mental states (e.g. beliefs, thoughts, feelings, or desires). People with ASPD show impaired recognition of basic emotions [35], impaired capacity to link mental states to behaviour [36, 37], and difficulty with perspective-taking problems and in reading others’ mental states [38–43]. They perform far worse than controls on subtle tests of mentalizing [44, 45]. These observations are consistent with a number of theories of antisocial behaviour [46, 47], including the deficit theory in the mentalizing literature, as well as the mentalizing model of antisocial behaviour, which is premised on the dysfunction of the attachment system that then temporarily inhibits affect regulation and mentalizing abilities [48–51]. Antisocial behaviour and violence tend to occur when an understanding of others’ mental states is developmentally compromised (fragile) and prone to being lost when the attachment system is activated by perceived threats to self-esteem, such as interpersonal rejection or disrespect [52]. Normally, mentalizing (i.e. envisioning the subjective state of the victim) prevents interpersonal violence [53]; this means that individuals with vulnerable mentalizing capacities can be behaviourally volatile in moments of interpersonal stress. Supporting the capacity to identify others’ emotions and intentions may not only assist social functioning but also reduce the risk of antisocial behaviour. Indeed, mentalizing has been shown to be a protective factor against aggression in people with violent traits [39]. Encouraging mentalizing has been shown to reduce school violence [54, 55], and MBT has demonstrated success in treating symptoms of impulsivity in individuals with comorbid BPD and ASPD [56]. Other studies of forensic patients with personality disorder have found that participants’ views of the processes by which therapeutic changes occurred tended to identify realizations that in turn reflected improved mentalizing [57, 58].

Recognizing the therapeutic potential of MBT for ASPD, the developers have adapted MBT specifically for individuals with ASPD, in line with the NICE guideline recommendation that interventions for ASPD should be geared to enabling individuals to better examine their own states of mind and understand others’ minds, and to behave more prosocially [5]. MBT-ASPD is a complex psychological intervention delivered using a combination of group and individual sessions [53, 59–61], which aim to enhance mentalizing by helping participants to develop metacognitive understanding of their difficulties with violence and achieve control over their aggression by addressing relevant drivers such as interpersonal misattributions (by improving interpersonal understanding through cognitive and affective identification with the experiences of others) and emotion dysregulation (by facilitating controlled and conscious mentalizing rather than automatic, non-reflective, non-conscious mentalizing).

### Aims

The primary aim of the Mentalization for Offending Adult Males (MOAM) trial is to address the following research question: is probation as usual (PAU) supplemented with MBT more effective and cost-effective than standard PAU alone for reducing aggressive antisocial behaviour in male offenders under community supervision who meet DSM-IV diagnostic criteria for ASPD? As there is currently poor characterization of the primary and secondary care needs of people with ASPD, the trial will aim to investigate the impact of MBT on a range of health-related and behavioural outcomes, some of which are of interest irrespective of any diagnosis (e.g. quality of life) and some of which are particularly pertinent to ASPD and its symptoms (e.g. offending, violence, substance use, remission from categorically defined ASPD status), and to examine the mediators and moderators of these outcomes. The trial will also aim to establish the relative cost-effectiveness of MBT and PAU, taking into account costs incurred and the return on investment across health services, social services, the criminal justice sector, and voluntary sector services during the 24-month period following randomization. Additionally, the trial will aim to analyse offender referral information to identify trends, calculate the size of the population likely to benefit from an intervention for ASPD, map outcomes from current interventions, and make recommendations about treatment groupings and targeting of services. Finally, the trial aims to generate and disseminate data that support policymakers, service commissioners, and service providers in making evidence-based decisions about planning and delivering services for offenders with ASPD on probation and in improving the management of mental health needs of offenders with ASPD in the community.

## Methods

### Trial design

The MOAM trial is a pragmatic, multi-site RCT comparing MBT with PAU for adult males meeting DSM-IV diagnostic criteria for ASPD [62], who are under community probation supervision. The design is a single-blind (with researchers being blinded) RCT of a 1-year programme of MBT-ASPD, which comprises weekly group sessions (75 min) and monthly individual sessions (50 min), compared with the usual services provided by probation (i.e. PAU) for this client group. Typically, PAU can include a variety of treatments, ranging from those that directly address personality disorder (e.g. schema-focused therapy) to those that address more specific criminological characteristics (e.g. anger management programmes, substance misuse interventions, domestic violence interventions), although in some services there are no targeted treatment options currently available for participants with a diagnosis of ASPD. This trial is unique among studies evaluating interventions for antisocial behaviour in that it is a full RCT with a large sample size, which targets ASPD as the primary diagnosis, and which differentiates ASPD from other mental health conditions.

### Ethics

The study protocol was approved by the London – South East Research Ethics Committee (reference number 14/LO/1696) and the National Offender Management Service (reference number 2014-315). Research and development approval has been sought and obtained for each trial site by the relevant NHS Trust and NPS lead in each geographical area. The trial sponsor, University College London, played no part in study design; collection, management, analysis, and interpretation of data; writing of the report; and the decision to submit the report for publication.

### Study setting

This study involves two UK statutory services—the NPS in partnership with NHS Trusts—at 13 sites. The MBT clinical services, like other National Offender Personality Disorder community services, were designed to be delivered through existing community partnerships between Probation Trusts and health service providers. Sites in England and Wales were invited to bid for the clinical services in 2014, prior to the commencement of the RCT in 2016. The National Offender Personality Disorder Programme covers England and Wales, but not Scotland or Northern Ireland, hence the restriction of sites to England and Wales. Providers needed to already be part of the Offender Personality Disorder Pathway Strategy with an existing contract in place between NHS England and the Probation Trust to deliver the community service specification in addition to a subcontract with a health service provider. Services were also selected on the basis of (a) geographical spread across England and Wales, (b) demographic representativeness (urban vs rural), and (c) availability of participants for recruitment into the trial (favouring somewhat larger services). The 13 sites comprise the following: NPS London and the Tavistock and Portman NHS Foundation Trust in conjunction with Barnet, Enfield, and Haringey Mental Health NHS Trust; NPS London and the South London and Maudsley NHS Trust; NPS Lincolnshire and the Lincolnshire Partnership NHS Foundation Trust; NPS Merseyside and the Merseyside Care NHS Trust; NPS Devon & Cornwall and the Devon Partnership NHS Trust; NPS London and the East London NHS Foundation Trust; NPS London and the Oxleas NHS Foundation Trust; NPS Lancashire and the Lancashire Care NHS Foundation Trust; NPS Staffordshire and West Midlands and the South Staffordshire and Shropshire Healthcare NHS Foundation Trust; NPS Nottinghamshire and the Nottingham Healthcare NHS Foundation Trust; NPS Gloucestershire and the Avon and Wiltshire Mental Health Partnership NHS Trust; NPS Wales and Hywel Dda University Health Board; and NPS West Yorkshire and Leeds & York Partnership NHS Foundation Trust.

### Participants

A total of 302 participants will be recruited, with approximately half of the consecutive qualifying cases being randomized to MBT and the other half to PAU. The unit of randomization is the individual participant.

Participants will be identified from a pool of offenders subject to statutory supervision by the NPS and assessed as having indications of personality disorder according to the Community Personality Disorder Pathways Service Specification of the Offender Personality Disorder Pathway Strategy following case identification, consultation, and formulation.

### Eligibility criteria

Inclusion and exclusion criteria have been carefully selected on the basis of previous research and clinical experience to provide an appropriate balance between homogeneity (to ensure that the treatment under investigation is appropriately aimed toward the diagnostic needs profiles of those within the trial) and heterogeneity (to accurately reflect the mix of antisocial offenders under the management of community probation and to ensure that the results of the trial are generalizable to the wider population of people diagnosed with ASPD) within the sample, as well as to enable comparability with other trials investigating the treatment of aggression and of antisocial behaviour [25, 63–65].

All participants will meet the following inclusion criteria:

- Male and aged 21 years or over
- Subject to statutory supervision by the NPS
- At least 6 months remaining of their licence or community sentence
- Fulfilling DSM-IV diagnostic criteria for ASPD (assessed by the Structured Clinical Interview for DSM-IV (SCID) Axis II Disorders (SCID-II)) [66]
- Score of at least 15 on the Overt Aggression Scale – Modified (OAS-M) [67]
- Adequate English language and cognitive capacities to participate in informed consent and group therapy.

The exclusion criteria applied will be minimal and are as follows:

- Serving a conviction for child sexual offences
- Primary diagnosis of a psychotic disorder or neurodevelopmental disorder.

### Sample size and power calculation

The sample size (*N* = 302) has been chosen to have 90% power, at a two-sided 5% significance level, to detect a significant difference between groups when the change in OAS-M score is on average 10 points less in the MBT arm (with a standard deviation of 20) at the primary endpoint 12 months after randomization. Hollander et al. [68] reported the pooled standard deviation of the change in OAS-M score from baseline to 10-week follow-up as being just over 9 for the pharmaceutical intervention assessed in that study. Due to the longer follow-up period of the present trial and the non-pharmaceutical nature of the planned intervention, we have chosen to increase the standard deviation used in our power calculation to 20. To take into account potential clustering by clinician in the MBT arm, we assume the intraclass correlation coefficient to be 0.05. In this case, using established methods to take account of clustering [69], 95 participants per arm will give 90% power. We anticipate attrition and dropout to be 37%, based on several sources including our own preliminary work and several larger data sources (a meta-analysis of attrition rate in offender treatment literature [70], a systematic review of non-completion of treatment for personality disorder [71], and an empirical evaluation of treatment disengagement in offenders with personality disorder [72]). To account for this loss, initial recruitment of 151 participants per arm is anticipated to result in 95 participants remaining per arm after attrition and dropout. Our target sample size is therefore 302 participants, which will give sufficient power to detect a medium effect size of 0.5 change in participants’ OAS-M score.

Demographic and clinical details from those refusing randomization will be retained to explore whether the trial population is representative. To assess treatment acceptability, we will compute the proportion of referred individuals who never attended, the number of sessions attended, and the number who drop out of treatment or request to be referred to other treatments. All participants who do not formally withdraw from the study will be followed up on routine measures of outcome.

### Peer researchers

A crucial aspect of this trial design is the use of peer researchers. This approach enables individuals with similar key criteria to the participant group to play an active role in the research process, by taking on the role of researchers. There are a number of potential benefits associated with using peer researchers, including the increased likelihood of accessing people and topics that traditional research and service staff may not be able to reach [73] and the potential to enhance the accuracy and validity of participant data through peer researchers being better placed to put participants at ease, thus stimulating free discussion and more open dialogue [74]. Peer researchers may also reduce the power differentials between researchers and participants, thereby facilitating trust and rapport and promoting honest disclosure of sensitive information [75]; this is particularly important in our target population, given that those involved in the criminal justice system often have an entrenched distrust of authority. In this study, peer researchers with lived experience of the criminal justice system, provided by the charity User Voice, will work alongside traditional research assistants under robust supervision arrangements to conduct baseline and outcome assessments. Peer researchers will be expected to have successfully reintegrated into society and have prior experience of an engagement role.

### Recruitment and baseline procedures

**Fig. 1:**
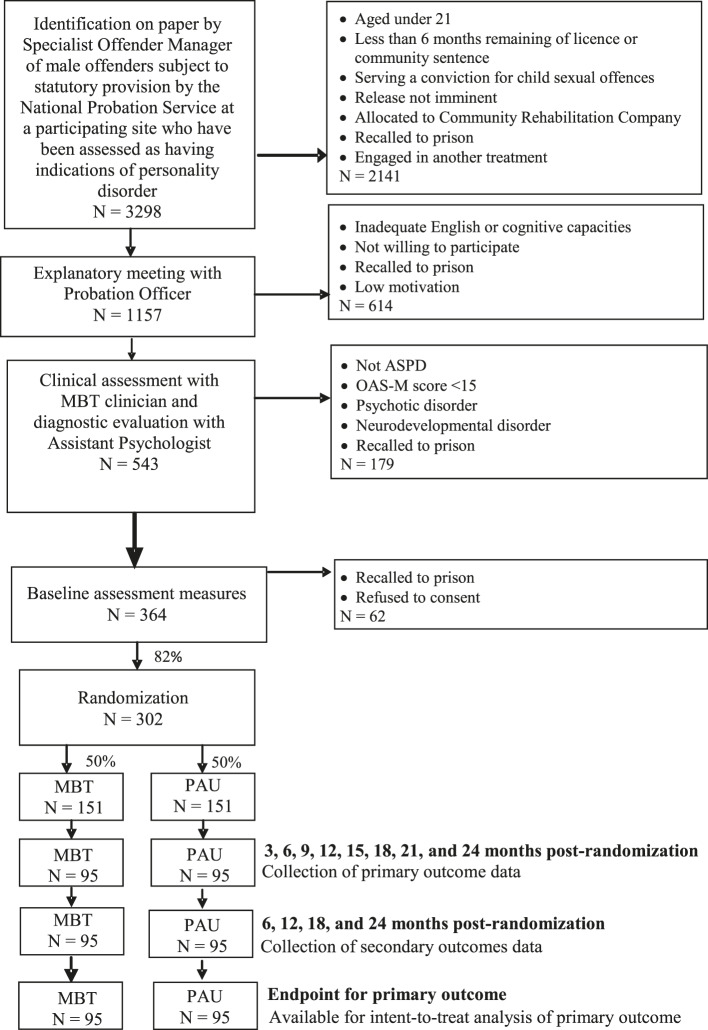
Expected flow of participants from recruitment through to the end of the study

Figure 1 shows the expected flow of participants from recruitment through to the end of the study, based on observed screening and referral data to date. In addition to aspects that apply to recruitment for any trial (e.g. the clear application of eligibility criteria, a standard procedure for obtaining informed consent), recruitment for this trial will be especially sensitive to the community probation context and will be based on effective partnerships with referral agencies and strong relationships with the participants. Consequently, the trial team has developed strong collaborative relationships with the MBT team at each site to achieve the high levels of accrual necessary to ensure sample comparability and reasonable generalizability.

We have developed a multiple gating procedure for recruitment. Decisions about eligibility for the trial will be made at three key points: (1) by the referring agencies (i.e. individual Offender Managers across each site) following a discussion with the Specialist Offender Manager (SOM) in the MBT team to identify ostensibly suitable potential participants; (2) through an explanatory meeting between the offender, the MBT team, and the research team following the initial referral, to explain the research trial, invite participation, and answer any questions about the clinical services potentially available to the participant; (3) as a result of discussions between the MBT clinicians, Assistant Psychologist, and Research Manager following an in-depth clinical assessment and formal diagnostic evaluation, to screen out offenders who do not meet the eligibility criteria.

Experience suggests that each of these screens tends to reveal different criteria for ineligibility, and their use in combination minimizes the (considerable) effort of recruitment. For example, the SOM tends to identify and screen out cases whose offending history includes child sex offences, whereas discussions between the MBT and research teams and the potential participant most commonly identify and screen out individuals who are insufficiently motivated to engage with group treatment. The clinical assessment reveals offenders who are ineligible due to their lack of suitability for tolerating a group setting or psychotherapeutic treatment in general, and the formal diagnostic evaluation is necessary to confirm the individual’s psychiatric diagnosis.

#### Screen 1

The SOMs in the MBT teams will work across each of the 13 sites, taking referrals from Offender Managers who will put forward individuals they deem agreeable to being approached about participation and who have adequate English and cognitive capacities to participate in informed consent and group therapy. SOMs will be available to discuss cases that potentially meet the eligibility criteria for MBT. In addition, the SOM and Assistant Psychologist at each site can directly screen for suitable cases by reviewing their caseload against the inclusion and exclusion criteria, before approaching the respective Offender Manager to make a referral.

The decisions made by the SOM constitute the first screen. A standard referral form for each locality is used in liaison with the research team, including specific information pertaining to inclusion and exclusion criteria.

#### Screen 2

Following acceptance of the referral by the MBT team, a meeting will be arranged between the MBT and research teams and the offender, to inform the offender about the trial and the opportunity to participate. The discussions at this meeting include what the MBT intervention would entail and the alternative PAU path (the best available alternative treatment at that locality; see below) should the offender not be randomized to receive MBT. Offenders will be provided with participant information sheets that have been co-designed with input from ex-offenders to make them as useful and accessible as possible. Offenders will be given a minimum of 24 h to consider participation.

#### Screen 3

If the offender decides to participate following the explanatory meeting, an in-depth clinical assessment and formal diagnostic evaluation will be arranged. MBT clinicians will conduct history-taking, administering the SCID-II psychosis screen, and ascertaining the offender’s ability to engage in psychotherapeutic treatment and tolerate a group setting.

Consent forms will be signed by both the offender and the site’s lead MBT clinician, who will assess the offender’s capacity to provide informed consent. Consenting to the trial includes providing permission to access police records, remaining in effect for 2 years, and health records, remaining in effect for 5 years.

The site’s Assistant Psychologist will administer the SCID-II screen and the OAS-M. If all of the inclusion criteria and none of the exclusion criteria are met, the participant will progress to the next stage of the research process to complete the baseline measures.

#### Baseline measures

For offenders who sign the consent forms, the researcher will administer pre-randomization questionnaires and measures to be completed during this contact (i.e. before group assignment). When all the instruments have been completed and eligibility for the trial has been confirmed, randomization will be performed and details communicated to the referrer, MBT team, and participant within 48 h.

#### Randomization and procedures to minimize bias

Following consent and baseline measures, a trial identification number will be assigned to eligible consenting participants and a recruitment ‘dam’ created behind which a participant pool will be built up, from which participants will be randomized to enable MBT groups to start. Once groups have started, sequential assignment will allocate participants to MBT or PAU in a 1:1 ratio. Randomization will be undertaken off-site, using a dynamic adaptive allocation algorithm [76] accessed by a secure web portal to the system held at NWORTH Clinical Trials Unit (CTU) and maintained by a statistician independent of the analysis and research teams to ensure blinding. Stratification will be by site, age (21–25; 26–39; 40+ years), probation order type (Community Sentence; On Licence Post-Prison), and time remaining on probation (less than 12 months; 12 months and over). Treatment site is included in the minimization stratification to control for differences between sites. To minimize bias that could arise from knowledge of treatment allocation, researchers will be blind to participants’ treatment. The allocation to treatment arms will not be modified unless the local principal investigator, with the agreement of the clinical supervisor or the Trial Steering Committee (TSC), identifies severe risks of adverse events (homicide, suicide, serious injury, etc.) associated with the experimental treatment. Experimental treatment will be discontinued if the participant breaks the conditions of probation and is recalled to serve the remainder of their prison sentence for a time which prevents any further attendance or if the participant commits further offences and receives a custodial sentence for the fresh conviction that is longer than the remaining time of treatment.

### Planned interventions

Implementing standard PAU or PAU supplemented with MBT will not require alteration to usual pathways (including use of any medication), and these will continue for both trial arms.

#### PAU

Participants randomized to receive PAU will remain under the supervision of the NPS for the duration of their licence or community sentence. PAU is a well-specified and stringently monitored standard procedure used in the management of offenders under community supervision. It consists of regular contact with a designated Offender Manager for the duration of the offender’s licence or sentence, and includes referral for additional interventions as indicated in Ministry of Justice guidance. PAU participants will be free to be referred by their Offender Manager for any locally available treatment (except MBT) designed to reduce reoffending (e.g. anger management programmes, substance misuse interventions). Provision of these interventions is not uniform across the UK but dependent on local availability; in some areas, there may be no targeted interventions available. Unlike MBT, none of the interventions that participants may receive under PAU involve targeting the capacity to envision mental states more accurately. Details of services accessed under PAU will be documented by the SOM to facilitate economic data collection and evaluation. Additional site-specific strategies (e.g. separate supervision groups) will ensure that MBT principles and practice do not directly influence the management of participants randomized to PAU. PAU will last for 12 months, after which participants who still have time remaining on their licence or community sentence will remain under the supervision of the NPS for the duration. Participants whose licence expires during the trial period may remain under the NPS on a voluntary basis to complete the 12-month period.

#### PAU supplemented with MBT-ASPD

The treatment intervention is a 1-year programme of group and individual sessions of MBT-ASPD. Participants randomized to MBT-ASPD receive weekly group therapy for 75 min and monthly individual therapy for 50 min. Each MBT-ASPD group is run by two trained MBT clinicians and contains a maximum of eight participants, using an ‘open’ rolling entry group, with new participants joining as others leave. The group process enables participants to challenge each other about their understanding of current self-identified critical interpersonal experiences and focuses particularly on elaborating the mental state background of interactions characterized by conflict that might normally trigger aggression. The content of the group therapy sessions is steered by the clinicians toward encouraging the participants to talk about their mental states related to recent violent incidents by linking their actions to a broader range of their current subjective and emotional experience (i.e. to ‘mentalize’ them). A monthly 50-min individual session provides an opportunity for participants to address personal issues that have occurred in the group. Many participants find it more difficult to talk in front of other members of the group about the relationship difficulties they experience in their external lives, and may use the individual sessions to do this too. Both group and individual sessions focus on identifying thoughts and feelings associated with aggressive impulses, with particular emphasis on understanding emotional cues, recognizing emotions in oneself and others, understanding others’ experiences in relation to one’s own, and clarifying threats of loss to mentalizing in the context of life experiences, both currently and historically.

All participants randomized to this arm of the study have an allocated clinician. Staff providing MBT sessions are chartered health professionals with a core clinical training background (e.g. clinical psychology, nursing, medicine) who have undergone an MBT training course. Where possible, recently trained clinicians are paired with an experienced clinician to provide group MBT.

The evaluation of the adequacy of protocol delivery and adherence to the model will include treatment integrity measures based on the MBT Adherence and Competence Scale [77], applied to video recordings of a random sample of sessions. This instrument enables coders to rate therapist activity and appropriateness in five key domains of MBT with ratings on Likert scales supported with anchored descriptors. As clinicians will be specially trained for the trial, it is critical to separately evaluate both clinician adherence and level of clinician competence, which will be based on the independent rating of randomly selected session recordings. The scale measures both the frequency of therapists’ actions (adherence) and the quality of the delivery of those actions (competence). Adherence refers to the number of items used within a domain and their frequency of use; quality refers to the clinician’s demonstration of how the interventions are delivered in terms of (a) expertise, competence, and commitment; (b) timing; (c) taking account of the context and content of the session; (d) matching the mentalizing state of the patient; (e) responding to where the patient appears to be; and (f) an extensiveness component in terms of items used when working in the domain. A definition of skill/quality is given for each domain.

Clinicians with average scores above 3.5 will be considered to have achieved acceptable levels of adherence and competence. Adherence will be monitored on a monthly basis through clinical supervision sessions using the MBT Adherence and Competence Scale, based on independent rating of randomly selected session video recordings, to ensure that clinical staff are consistently on-model.

### Assessments and outcome measures

To maximize the validity of the outcome evaluations, assessments will be made across multiple domains using multiple methods and sources.

#### Primary outcome

The primary outcome is the frequency of aggressive antisocial behaviour as measured by an adapted version of the OAS-M and is collected every 3 months. The OAS-M provides information to establish frequencies of verbal aggression and physical aggression against objects, against others, and against the self. The OAS-M is a robust semi-structured interview and is the most widely used measure of aggression in trials and clinical studies in this area. It has been used with many different outpatient populations, including those with Cluster B personality disorders. It is one of the only measures of aggression that assesses actual aggressive behaviour, and its format offers several advantages over most available instruments for assessing anger and aggression, including allowing the probing of vague and inconsistent answers [67]. It is therefore well suited to accurate evaluation of aggressive behaviour and of change over time, both as a research instrument and as a complement to many of the current self-report measures. In agreement with the developer of the measure, the OAS-M, adapted for the current study, uses a weighted scale taking the number of reported endorsed items and multiplies them in accordance with the severity of each item. Aggression is the primary outcome in this study as it is the key characteristic of ASPD, the primary driver of interpersonal violence, and a key contributor to health status [78, 79]. The primary endpoint is at 12 months post-randomization. As a secondary outcome, the adapted OAS-M is collected at other time points (3, 6, 9, 15, 18, 21 and 24 months).

#### Secondary outcomes

The domains that the investigators consider key to the intervention are violence, anger, offending behaviour, impulsivity, alcohol and substance misuse, self-harm and suicidality, general health and wellbeing, service satisfaction and engagement outcomes, and remission in ASPD diagnostic status. These secondary outcomes will be assessed by using objective and self-report measures. Estimates of the population at risk resource use data relevant to conducting a comprehensive economic evaluation will be collected alongside these outcomes, as well as data on variables associated with putative mechanisms of change.

#### Objective measures

Objective outcomes of health service use will be collected from record linkage using participants’ NHS numbers at 6-monthly intervals during the intervention and the follow-up period until the 24-month follow-up point.

Objective outcomes of offending behaviour, including withdrawal of licence, arrests, and reconvictions, will be collected from police computer records at 6-monthly intervals, for the 6 months before randomization and during the intervention and follow-up period until the 24-month follow-up point. The number of records of offending behaviour (count data) will be obtained, and 6-month periods free of any offending behaviour will also be recorded (binary data). Data will be obtained from participants’ records on nDelius (the national public sector offender case-management system) and the Police National Computer database; these records detail information on breaches of licence conditions, police contacts, charges, court appearances, criminal orders, arrest rates, and frequency, severity, and type (violent/non-violent) of offending behaviour.

These objective outcomes of health service use and offending behaviour will be available regardless of loss to follow-up; this will allow the use of multiple imputation techniques. If loss to follow-up is considerably different between arms and the primary outcome is significant, we will perform a sensitivity analysis to the impact of missing data not being missing at random, as described by White et al. [80].

#### Self-report measures

Researchers will administer pre-testing questionnaires during the initial contact with participants after they have given consent to participate in the trial, before group assignment (as described above). Follow-up assessments will be made at 3-month intervals for the primary outcome measure and for secondary outcome measures assessing violence, intimate partner violence, and anger; at 12-month intervals for the secondary outcome measures assessing categorically defined ASPD and rates of desistance; and at 6-month intervals for all other secondary outcome measures. Assessments will continue for the duration of the 12-month treatment and for 12 months post-treatment. Self-report measures of violence, offending behaviour, substance misuse, self-harm, and suicidality, as well as general health and wellbeing, service satisfaction, and engagement, will be collected.

*Violence* will be measured by the MacArthur Community Violence Screening Instrument [25, 81]. This measure assesses the presence, severity, and frequency of violent behaviours and is one of the few instruments available to differentiate between general aggression and specific interpersonal violence [82].

*Intimate partner violence* will be measured by the Short Form of the Revised Conflict Tactics Scales. This measure includes scales to measure physical assault, psychological aggression, and sexual coercion against a partner in a dating or marital relationship.

*Anger* will be measured by the State-Trait Anger Expression Inventory-2 (STAXI-2) [83, 84]. The STAXI-2 is a psychometrically robust questionnaire [85] designed to assess the experience, expression, and control of anger, which commonly drives aggression.

*Alcohol use*, which has been strongly associated with aggression and offending [86], will be measured by the Alcohol Use Disorders Identification Test (AUDIT) [85, 87]. The AUDIT is widely used as an alcohol use screening measure. It assesses three aspects of drinking: quantity and frequency, indicators of dependence, and adverse consequences suggesting harmful use.

*Drug use*, which is similarly linked to aggression and offending [88], will be measured by the Drug Use Disorders Identification Test (DUDIT) [89], which assesses frequency of use, indicators of dependence, and adverse consequences suggesting harmful use. The DUDIT has several advantages over other instruments, including a brief administration time (5 min) and its focus on recent use and consequences.

*Self-harming behaviours*, an indicator of impulsivity and a target of MBT, will be measured by the Self-Harm Inventory [90], a widely used measure that yields a severity of self-harm score based on the frequency of a wide range of impulsive behaviours.

*Suicidal behaviour*, a further complication of offending behaviour with ASPD [91], will be measured by the Suicidal Behaviours Questionnaire-Revised [92]. This instrument assesses four different dimensions of suicidality, including frequency of suicidal ideation and threat of suicide attempt.

*Health-related quality of life* will be measured by the EQ-5D-5L [93, 94]. The EQ-5D-5L is a standardized instrument for use as a measure of health outcome that is applicable to a wide range of health conditions and treatments. In this study, it will be used to provide a generic measure of health for both clinical and economic appraisal.

*Mental health status* will be measured by the Symptom Checklist-90 – Revised (SCL-90-R) [95]. The SCL-90-R assesses psychological distress in terms of nine primary symptom dimensions, including depression, anxiety, hostility, and paranoid ideation—all potential drivers of aggression.

*Personality dysfunction* will be measured by the Personality Inventory for DSM-5 – Brief Form (PID-5-BF) [96]. This 25-item personality trait assessment scale assesses negative affect, detachment, antagonism, disinhibition, and psychoticism and will provide a means of assessing change in the trait domains that are most prominent in ASPD.

*Categorically defined ASPD* will be measured by the ASPD module of the SCID-II, to assess whether MBT produces remission in ASPD diagnostic status.

*Mentalizing capacity* will be evaluated by the 16-item Brief Reflective Functioning Questionnaire [97] to assess whether MBT’s proposed mechanism of change is in line with any symptomatic improvements.

*Rates of desistance* will be measured by the Redemption and Condemnation Self-Narrative Scale version 2 to assess whether MBT increases rates of desistance in the participants.

#### Clinician-rated measures

Clinicians (therapists, Assistant Psychologists, and Offender Managers) will use the Service Engagement Scale [98] to determine the level of engagement of participants, as rated on four subscales: availability, collaboration, help-seeking, and treatment adherence.

#### Economic evaluation

Health economic analysis will be conducted by King’s Health Economics at the Institute of Psychiatry, Psychology and Neuroscience, King’s College London, and will explore the relative costs and cost-effectiveness of MBT+PAU versus PAU. Although there is a preference for economic evaluations carried out on NHS services to take an NHS and personal and social services cost perspective, as recommended by NICE [99], in this case our primary analysis will take a wider societal perspective, including all healthcare, social and personal services, voluntary sector services, costs to the criminal justice sector, including probation, and the costs resulting from any crimes committed. The wider perspective is relevant and valid here because the interventions being compared are delivered in a criminal justice setting and the hypothesized impact will fall across both healthcare and criminal justice. A secondary analysis will consider the NHS/personal and social services costs only. Data on MBT contacts will be collected directly from sites, to avoid participants revealing their group allocation to the researchers. Data on the use of all other services, including PAU, will be collected in interviews using the Secure Facilities Service Use Schedule [100]. This instrument has been adapted to the current study population to ensure comprehensive coverage and face validity. Information on criminal activity will be extracted from police records. The cost of the trial interventions will be calculated through a detailed micro-costing (or bottom-up) approach using standard costing methodology [101], which will involve estimation of the indirect time spent on individual cases, including preparation, meetings, telephone calls, and supervision, as well as detailed recording of direct face-to-face contact. Other unit costs will be sourced from routine sources for the unit cost year 2017–2018 [102–104].

#### Follow-up assessment

Follow-up assessments will be conducted at 3-month intervals for 24 months post-randomization. Primary outcome measures will be collected every 3 months and secondary outcome measures every 6 months. Table 1 shows a detailed outline of the planned measures at each follow-up point throughout the trial.

**Table 1:** Schedule of assessment administration for a randomized controlled trial of mentalization-based treatment for antisocial personality disorder

	Study period										
	Enrolment	Allocation	Post-allocation								
			T1	T2	T3	T4	T5	T6	T7	T8	T9
**ENROLMENT**											
Eligibility screen	x										
Informed consent	x										
Allocation		x									
**INTERVENTION**											
MBT			x	x	x	x	x				
PAU			x	x	x	x	x				
**ASSESSMENTS**											
**Questionnaires—participant**											
Overt Aggression Scale – Modified			x	x	x	x	x	x	x	x	x
EQ-5D-5L			x		x		x		x		x
Symptom Checklist-90–Revised Short Form			x		x		x		x		x
Alcohol Use Disorders Identification Test			x		x		x		x		x
Drug Use Disorders Identification Test			x		x		x		x		x
State-Trait Anger Expression Inventory			x	x	x	x	x	x	x	x	x
MacArthur Community Violence Screening Instrument			x	x	x	x	x	x	x	x	x
Short Form of the Revised Conflict Tactics Scales			x	x	x	x	x	x	x	x	x
Self-Harm Inventory			x		x		x		x		x
Suicidal Behaviours Questionnaire – Revised			x		x		x		x		x
Secure Facilities Service Use Schedule			x		x		x		x		x
Personality Inventory for DSM-5-Brief Form			x		x		x		x		x
SCID-II, ASPD Module			x				x				x
Brief Reflective Functioning Questionnaire			x		x		x		x		x
Redemption and Condemnation Self-Narrative Scale version 2							x				x
**Questionnaires—clinician**											
Service Engagement Scale					x		x		x		x
National Probation Resources, Evaluation and System Schedule			x		x				x		x
**Police and health data**											
Police records			x		x		x		x		x
Health records			x		x		x		x		x

### Data management

#### Collection

Outcome measures data will be recorded using the Patient Owned Database (POD), an internet-based high-security data collection and management software. POD automatically scores and transmits the data to the data-analytic site without human interference, reducing the risk of data entry error. Once entered, the data will be exported and cleaned by the CTU ready for analysis by the trial statistician. In line with a data sharing agreement with the NPS and UCL Data Protection Policy, participants’ personal data will be collected and securely stored in password-protected files only accessible by members of the research team during the course of the study. At the end of the trial, all data will be archived in a safe and secure off-site location.

#### Auditing

The quality of data will be routinely monitored by the Research Manager throughout the duration of the trial. In addition, data quality will be monitored quarterly by an independent audit group, which will be responsible for reviewing data collection and completeness rates.

### Statistical analysis

All planned statistical analyses will be specified in a Statistical Analysis Plan that is agreed prior to unblinded information being made available to the trial statistician. Primary analyses will be on an intent-to-treat basis, with per-protocol (all participants who were randomized, who met inclusion/exclusion criteria, and who attended at least 35% of planned meetings with their parole officer) analyses undertaken as secondary. The characteristics of the treatment groups will be described at baseline. Preliminary analysis will investigate the pattern of missing follow-up data.

#### Primary outcome

The primary outcome (OAS-M) at the various follow-up times will be analysed using a hierarchical mixed-effects linear regression with baseline OAS-M, length of sentence for index offence, and the stratification factors used in the randomization (except for site) as fixed effects. Patient ID and site will be included as random effects. A treatment effect parameter for each follow-up time will be included, although the primary one of interest will be treatment effect at 12 months. Two-sided *p* values less than 0.05 will be classed as significant.

#### Secondary outcomes

For the secondary outcomes, we will use mixed-effects linear regression for continuous outcomes, mixed-effects logistic regression models for binary outcomes, and mixed-effects Poisson models for count data such as number of offences. Missing outcome data due to dropout will be accounted for by the mixed-effects model under a missing-at-random assumption; missing covariates will be accounted for using multiple imputation.

#### Moderators of outcomes

The trial will evaluate the following as potential moderators for the primary and any secondary outcomes that are significantly different between arms: age, type of probation, length of probation, anxiety measured by the SCL-90-R, Axis II comorbidities measured by the SCID, p factor scores derived from a bi-factor analysis of the self-report outcome measures, baseline alcohol use measured by the AUDIT, and baseline drug use measured by the DUDIT.

Moderators will be tested by including interaction parameters in the model and testing them for significance.

#### Mediators of outcomes

We will test the following as mediators (if there is a significant difference between arms in the mediating variable): alcohol use (AUDIT score); drug use (DUDIT score); mood (scores on the anxiety and depression subscales of the SCL-90-R); mentalizing (Brief Reflective Functioning Questionnaire score).

We will also test the proportion of MBT planned sessions attended as a post-baseline effect modifier (as it is only collected in the MBT arm).

#### Economic evaluation

Mean total costs for the two groups at 24-month follow-up will be compared using a regression model with baseline costs as a covariate, an approach used despite the likely skewed distribution of the data because of the importance of the arithmetic mean in using the results for policymaking [105]. To test the robustness of the normality assumption, bootstrapped confidence intervals for the regression model will be also be calculated [106]. Quality-adjusted life years (QALYs) will be calculated from EQ-5D-5L health status using UK values for utility status [107] and assuming a linear change in utility over time. A cost-utility analysis will combine costs with the QALYs, a cost-effectiveness analysis will combine costs with aggressive behaviour and incremental cost-effectiveness ratios, and cost-effectiveness acceptability curves will be generated. Sensitivity analyses will include a test of the impact of the intervention costs on the cost-effectiveness results, and the impact of missing data, tested using multiple imputation of missing cost items.

## Oversight and monitoring

### Oversight committees

The study will be overseen by two oversight committees: the TSC and a Data Monitoring and Ethics Committee (DMEC). The TSC will be chaired by an independent expert and will be responsible for monitoring progress of the study and reviewing amendments to the trial protocol. Committee members will include experienced clinicians, commissioners of forensic mental health services, criminal justice professionals, and service user representatives. A group of clinicians with relevant experience in the area and expert trial statisticians will form the independent DMEC, which will be responsible for monitoring recruitment and follow-up rates across both arms of the trial, as well as serious adverse events (SAEs) and ethical concerns. Minutes from the DMEC will be shared with the TSC chair following each meeting, and the TSC will report directly to the funder.

### Adverse events

For this study, adverse events will be defined as immediate risk of harm to the participant or another person and will be collected after the participant has provided consent and enrolled in the study. Each event will be logged by the research team following contact with the participant and regularly reviewed by the Research Manager, Clinical Lead, and Chief Investigator. If an adverse event meets the criteria for a serious adverse event (SAE) between study enrolment and the 24-month data collection time point, the event will be logged and reported to the DMEC for review. SAEs occurring after a participant is discontinued from the study will not be reported unless the investigators feel that the event may have been caused by a study protocol procedure.

## Dissemination policy

The results of the study will be disseminated through publications in peer-reviewed journals as well as presentations, newsletters, and articles for the probation and prison system and third sector organizations working within the criminal justice system.

## Discussion

This study will address key gaps in the offending and health literature. To date, there have been very few high-quality trials with a sufficiently powered sample size that have evaluated therapeutic interventions for offenders with a primary diagnosis of ASPD. The MOAM trial is the first large-scale trial of treatment for offenders with ASPD in the community and has a number of strengths compared with other trials of interventions for antisocial behaviour. First, it is a definitive RCT, conducted in real-life community settings, at multiple sites with a representative geographical spread and a range of clinicians from diverse disciplinary backgrounds. Second, the sample size is sufficient to yield 90% power, even after accounting for a potentially high attrition rate. Third, its sample is selected on the primary diagnosis of ASPD as fully differentiated from other mental health conditions. Fourth, the trial design incorporates the innovative use of peer researchers as a strategy to enhance the accuracy and validity of participant data. Finally, the measures employed will enable us to look beyond recidivism and will capture the effects of MBT on a wide range of secondary health and behavioural outcomes, including diagnostic change of ASPD itself.

Aggressive antisocial behaviour has been chosen as the primary outcome for this trial, in light of it being a core feature of ASPD, an indicator of emotional wellbeing, and a frequent cause of grounds for arrest. A range of secondary outcomes have also been chosen in order to evaluate MBT’s possible impact on the wide range of symptomatology and health outcomes experienced by individuals with ASPD, including impulsivity, offending behaviour, alcohol and substance misuse, self-harm and suicidality, and general health and wellbeing. The MOAM trial will also provide accurate information about the services accessed under PAU. A comprehensive cost-effectiveness evaluation will be undertaken to examine both costs offset and costs saved in relation to having received MBT or PAU. Thus, the trial will provide unprecedented data not only on the clinical effectiveness and cost-effectiveness of MBT-ASPD, but also on the clinical effectiveness and cost-effectiveness of the usual services available to this group while on probation. Furthermore, the trial will analyse offender referral information to identify trends, calculate the size of the population likely to benefit from an intervention for ASPD, and make recommendations about treatment groupings and targeting of services.

Information to date suggests that the trial protocol detailed above is acceptable to all the sites and that recruitment, data collection, and training are feasible in all locations. Preliminary data on participant engagement indicate that, of participants randomized to MBT, an average of 55–65% attend group MBT sessions. Preliminary data indicate that one third of participants drop out at some point following randomization, entirely consistent with attrition rates reported in other studies involving treatment for offenders and patients with personality disorders [70–72]. For participants who drop out, the average length of engagement with the trial is 3–6 months; the most common reasons for dropout thus far are lack of motivation (58%), recall to prison (35%) and clinicians’ discretion due to repeated non-attendance of MBT sessions (7%).

The authors acknowledge that there is such a scarcity of evidence for effective interventions for ASPD that additional trials are greatly needed. Beyond the completion of the MOAM trial, the authors recommend further high-quality RCTs incorporating the following adaptations: minimal involvement of the intervention developers, to minimize bias; a female sample, to improve generalizability of results; longer-term follow-up, to determine whether differential effects are maintained; and investigation of the impact of differences in clinicians’ training, experience, and supervision, to determine minimal training and supervisory standards and competences for effective delivery of treatment.

## Trial status

The trial is in its fifth year. Sites were launched in four phases between January and September 2016, and recruitment ended in August 2018. Treatment and follow-up data collection are currently in progress. The protocol paper was submitted after the end of recruitment as the outcome frequency of aggressive antisocial behavior is measured at the last follow-up meeting 24 months post-randomization. Outcome data will be available 12 months post-randomization of the last participant.

*Trial protocol version:* v6.0, 2 December 2018.

## Data Availability

Not applicable.

## Abbreviations

ASPD: Antisocial personality disorder
AUDIT: Alcohol Use Disorders Identification Test
BPD: Borderline personality disorder
CTU: Clinical Trials Unit
DMEC: Data Monitoring and Ethics Committee
DUDIT: Drug Use Disorders Identification Test
MBT: Mentalization-based treatment
MBT-ASPD: Mentalization-based treatment for antisocial personality disorder
NICE: National Institute for Health and Clinical Excellence
NPS: National Probation Service
OAS-M: Overt Aggression Scale – Modified
PAU: Probation as usual
PID-5-BF: Personality Inventory for DSM-5–Brief Form
QALY: Quality-adjusted life year
POD: Patient Owned Database
RCT: Randomized controlled trial
SAE: Serious adverse event
SCID: Structured Clinical Interview for DSM-IV
SCL-90-R: Symptom Checklist-90 – Revised
SOM: Specialist Offender Manager
STAXI-2: State-Trait Anger Expression Inventory-2
TSC: Trial Steering Committee
