# Correction: Mentalization for Offending Adult Males (MOAM): study protocol for a randomized controlled trial to evaluate mentalization-based treatment for antisocial personality disorder in male offenders on community probation

**DOI:** 10.1186/s13063-025-08729-6

**Published:** 2025-03-13

**Authors:** Peter Fonagy, Jessica Yakeley, Tessa Gardner, Elizabeth Simes, Mary McMurran, Paul Moran, Mike Crawford, Alison Frater, Barbara Barrett, Angus Cameron, James Wason, Stephen Pilling, Stephen Butler, Anthony Bateman

**Affiliations:** 1 Research Department of Clinical, Educational and Health Psychology, University College London, London, UK; 2 Anna Freud National Centre for Children and Families, London, UK; 3 Portman Clinic, Tavistock and Portman NHS Foundation Trust, London, UK; 4 Institute of Mental Health, University of Nottingham, Nottingham, UK; 5 Centre for Academic Mental Health, Population Health Sciences Department, Bristol Medical School, University of Bristol, Bristol, UK; 6 Centre for Mental Health, Imperial College, London, UK; 7 School of Law, Royal Holloway, University of London, London, UK; 8 Institute of Psychiatry, Psychology and Neuroscience, King’s College London, London, UK; 9 National Probation Service London Division, London, UK; 10 Population Health Sciences Institute, Newcastle University, Newcastle Upon Tyne, UK; 11 MRC Biostatistics Unit, University of Cambridge, Cambridge, UK; 12 Psychology Department, University of Prince Edward Island, Charlottetown, Canada

**Correction: Trials 21, 1001 (2020)**

**https://doi.org/10.1186/s13063-020-04896-w**

Following the publication of the original article [1], we were notified that the primary endpoint was incorrectly described as being assessed at 24 months post-randomization. However, the correct time point for the primary endpoint, as outlined in the ethically approved study protocol document, is 12 months post-randomization.

This error has only come to light upon the trial's completion due to the length of time that has elapsed since ethical approval, trial registration, the development of the statistical analysis plan, and the publication of the study protocol.

The original article has been corrected.

## References

[1] Fonagy P, et al. Mentalization for Offending Adult Males (MOAM): study protocol for a randomized controlled trial to evaluate mentalization-based treatment for antisocial personality disorder in male offenders on community probation. Trials. 2020;21:1001. 10.1186/s13063-020-04896-w. 33287865 10.1186/s13063-020-04896-w PMC7720544

